# Ultrasound-guided, continuous erector spinae plane (ESP) block in minimally invasive thoracic surgery—comparing programmed intermittent bolus (PIB) vs continuous infusion on quality of recovery and postoperative respiratory function: a double-blinded randomised controlled trial

**DOI:** 10.1186/s13063-022-06726-7

**Published:** 2022-09-21

**Authors:** Aisling Ni Eochagain, Aneurin Moorthy, Áine O’Gara, Donal J. Buggy

**Affiliations:** 1 Division of Anaesthesiology & Perioperative Medicine, Mater Misericordiae University Hospital, Dublin, Ireland; 2 School of Medicine, University College, Dublin, Ireland; 3 Department of Anaesthesia and Pain Medicine, St James’s University Hospital, Dublin, Ireland

**Keywords:** Erector spinae catheter, Continuous infusion, Programmed intermittent bolus, Minimal invasive thoracic surgery, Quality of recovery, Chronic persistent surgical pain

## Abstract

**Background:**

Minimally invasive thoracic surgery (MITS) has been shown to reduce postoperative pain and contribute to better postoperative quality of life as compared to open thoracic surgery (Bendixen et al., Lancet Oncol 17:836–44, 2016). However, it still causes significant post-operative pain. Regional anaesthesia techniques including fascial plane blocks such as the erector spinae plane block (ESP) have been shown to contribute to post-operative pain control after MITS (Finnerty et al., Br J Anaesth 125:802–10, 2020). Case reports relating to ESP catheters have described improved quality of pain relief using programmed intermittent boluses (PIB) instead of continuous infusion (Bendixen et al., Lancet Oncol 17:836–44, 2016). It is suggested that larger, repeated bolus dose may provide superior pain relief, possibly because of improved spread of the local anaesthetic medications (Ilfeld and Gabriel, Reg Anesth Pain Med 44:285–86, 2019). Evidence for improved spread of local anaesthetic may be found in one study which demonstrated that PIB increased the spread of local anaesthetic medication compared to continuous infusions for continuous paravertebral blocks, which are another type of regional anaesthesia technique for the chest wall (Hida et al., Reg Anesth Pain Med 44:326–32, 2019). Similarly, regarding labour epidural analgesia, the weight of evidence is in favour of PIB providing better pain relief compared with continuous infusion (Onuoha, Anesthesiol Clin 35:1–14, 2017).

Since fascial plane blocks, such as ESP, rely on the spread of local anaesthetic medication between muscle layers of the chest wall, intermittent boluses may be particularly useful for this group of blocks. However, until recently, pumps capable of providing automated boluses in addition to patient-controlled boluses were not widely available. To best of our knowledge, there are no randomised controlled trials comparing continuous infusion versus intermittent bolus strategies for erector spinae plane block for MITS in terms of patient centred outcomes such as quality of recovery.

**Methods:**

This trial will be a prospective, double-blinded, randomised controlled superiority trial. A total of 60 eligible patients will be randomly assigned to receive an intermittent bolus regime of local anaesthetic vs a continuous infusion of local anaesthetic. The medication will be delivered via an ultrasound-guided erector spinae plane block catheter which will be inserted by an anaesthesiologist while the patient is under general anaesthetic before their MITS surgery begins. The primary outcome being measured is the Quality of Recovery (QoR-15) score between the two groups 24 h after surgery. Secondary outcomes include respiratory testing of maximal inspiratory volume measured with a calibrated incentive spirometer, area under the curve for Verbal Rating Score for pain at rest and on deep inspiration versus time over 48 h, total opioid consumption over 48 h, QoR-15 score at 48 h and time to first mobilisation.

**Discussion:**

Despite surgical advancements in thoracic surgery, severe acute post-operative pain following MITS is still prevalent. This study will provide new knowledge and possible recommendations about the efficacy of programmed intermittent bolus regimen of local anaesthetic vs a continuous infusion of local anaesthetic via an ultrasound-guided erector spinae plane catheter for patients undergoing MITS.

**Trial registration:**

This trial was pre-registered on ClinicalTrials.gov Identifier: NCT05181371. Registered on 6 January 2022. All item from the World Health Organization Trial Registration Data set have been included.

**Supplementary Information:**

The online version contains supplementary material available at 10.1186/s13063-022-06726-7.

## Administrative information

Note: the numbers in curly brackets in this protocol refer to SPIRIT checklist item numbers. The order of the items has been modified to group similar items (see http://www.equator-network.org/reporting-guidelines/spirit-2013-statement-defining-standard-protocol-items-for-clinical-trials/).

**Table:** 

Title {1}	Ultrasound Guided, Continuous Erector Spinae Plane (ESP) Block in Minimally Invasive Thoracic Surgery: Comparing Programmed Intermittent Bolus (PIB) vs Continuous Infusion on Quality of Recovery and Postoperative Respiratory Function: A Double-Blinded Randomised Controlled Trial.
Trial registration {2a and 2b}.	This trial was pre-registered on ClinicalTrials.gov Identifier: NCT05181371. Registered on 6 January 2022. All item from the World Health Organization Trial Registration Data set have been included. https://clinicaltrials.gov/ct2/show/NCT05181371
Protocol version {3}	Protocol version 2 as of 23/05/2022.
Funding {4}	Internal funding has been supplied for this trial from the divisions of Anaesthesiology at the Mater University Hospital and St. James’s Hospital. The trial has also received external funding (total of €10,000) from the European Society of Regional Anaesthesia (ESRA).
Author details {5a}	(1): Dr Aisling Ni Eochagain*: Locum Consultant Anaesthesiologist, Division of Anaesthesiology & Perioperative Medicine, Mater Misericordiae University Hospital, Dublin, Ireland; aislingnie@gmail.com. *Correspondence (2): Dr Aneurin Moorthy: Locum Consultant Anaesthesiologist/Research fellow, Division of Anaesthesiology & Perioperative Medicine, Mater Misericordiae University Hospital, Dublin, Ireland, School of Medicine, University College, Dublin, Ireland; aneurin.moorthy@gmail.com (4): Dr Áine O’Gara: Consultant Anaesthesiologist and Pain Medicine, Department of anaesthesia and pain medicine, St James’s University Hospital, Dublin, Ireland; aineogara@gmail.com (3): Professor Donal J. Buggy**: Consultant Anaesthesiologist, Division of Anaesthesiology & Perioperative Medicine, Division of Anaesthesiology & Perioperative Medicine, Mater Misericordiae University Hospital, Dublin, Ireland, School of Medicine, University College, Dublin, Ireland; donal.buggy@ucd.ie **Principal investigator
Name and contact information for the trial sponsor {5b}	Department Anaesthesiology & Perioperative Medicine, Mater University Hospital, Dublin, Ireland. Anaes@mater.ie office: Office: +35318032281
Role of sponsor {5c}	This is a hypothesis-driven, investigator-initiated trial. Funders played no role in the design of the study, data collection, analysis, interpretation of data or in the writing of the manuscript.

## Introduction

### Background and rationale {6a}

Minimally invasive thoracic surgery (MITS) is a surgical method used to perform lung surgery through small surgical incisions between the ribs and includes both video-assisted thoracic surgery (VATS) and robotic assisted thoracic surgery (RATS) [1, 2]. MITS has increased to almost half of all thoracic surgery in the past decade [3]. When compared to thoracotomy, MITS is associated with less pain, better shoulder function, earlier mobilisation, shorter LOS, better preservation of pulmonary function and better quality of life [3, 4]. Despite surgical advancements in thoracic surgery, severe acute post-operative pain following MITS is still prevalent [5].

In the past 5 years, the erector spinae plane (ESP) block has emerged as a novel regional anaesthesia procedure which has had some promising early results in attenuating this severe acute pain of MITS. In a recent RCT among MITS patients, single shot ESP block improved QoR-15 and reduced overall complications at 24 h compared with single-shot serratus anterior plane block [6]. Furthermore, our research group recently completed a randomised controlled trial comparing the efficacy of ESP catheters vs the gold standard paravertebral catheters for patients undergoing MITS and is currently under peer review [7].

Evidence for the improved spread of local anaesthetic with the use of programmed intermittent bolus techniques [8] have been described in studies of paravertebral blocks, which demonstrated that automated boluses increased the number of affected dermatomal levels compared to continuous infusions and could benefit patients requiring a wider extent of anaesthesia [9, 10]. Similarly, regarding labour epidural analgesia, the weight of evidence is in favour of programmed intermittent boluses [11]. Studies in this patient population have demonstrated the benefits of the programmed intermittent epidural bolus technique, including the use of less local anaesthetic and opioids, the occurrence of less breakthrough pain, improved patient satisfaction and potentially a lower incidence of both motor block and instrumental vaginal delivery [12, 13].

This study will provide new knowledge and possible recommendations about the efficacy of programmed intermittent bolus regimen of local anaesthetic vs a continuous infusion of local anaesthetic via an ultrasound-guided erector spinae plane catheter for patients undergoing MITS.

### Objectives {7}

Our main aim and corresponding hypotheses are outlined below:

We aim to complete a prospective, double blinded, factorial design, randomised controlled, superiority trial to test the hypothesis that programmed intermittent bolus techniques, as a maintenance mode for analgesia via erector spinae plane catheters, may provide better outcomes over traditional continuous infusion techniques, in terms of early recovery (QoR-15) at 24 h after MITS surgery.

### Trial design {8}

**Fig. 1:**
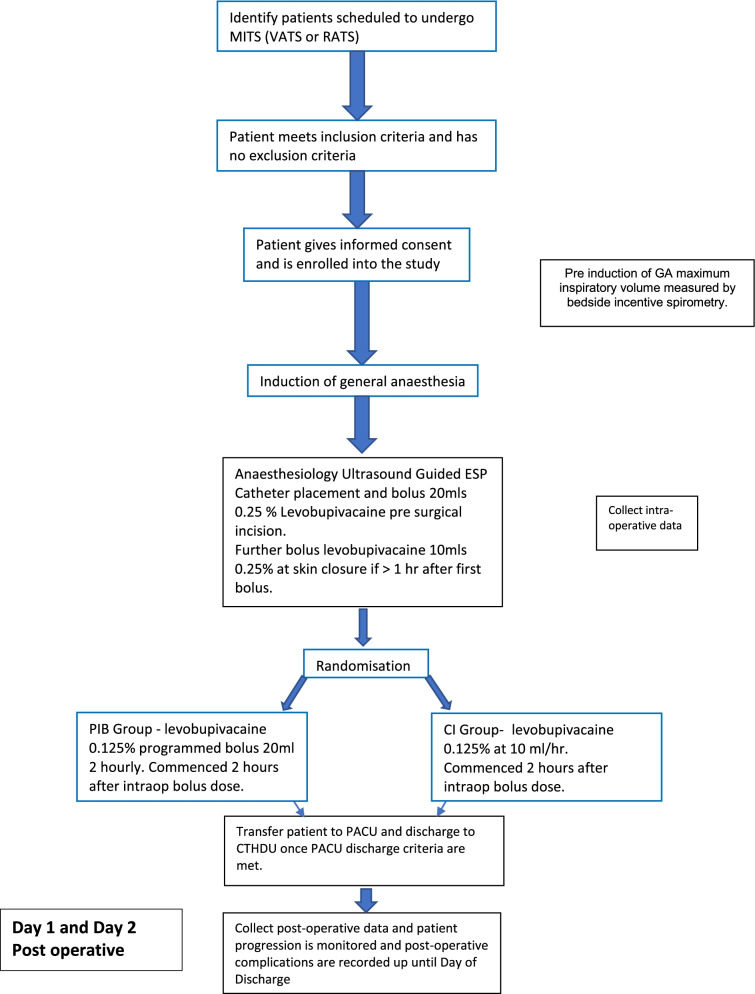
Study flow chart

We outline a research protocol for a double-blind, randomised controlled, superiority trial. Patients who enrol to this clinical trial will be randomly and equally (1:1) allocated into one of two intervention groups: programmed intermittent bolus (PIB) regime of local anaesthetic vs a continuous infusion (CI) of local anaesthetic via an ultrasound-guided erector spinae plane block catheter. Both groups will receive the same standardised pain relief protocol both during and after their surgery. Figure 1 illustrates the study flow chart. Recruitment commenced on June 2022, and it is expected to take between 9 and 12 months to complete.

## Methods: participants, interventions, and outcomes

### Study setting {9}

Ethical approval (Institutional Review Board; reference number 1/378/2292) has been granted for Mater Misericordiae University Hospital (MMUH), Dublin, Ireland, and recruitment has begun. In addition, we are currently seeking ethical approval for a second clinical site (St. James’s Hospital, Dublin, Ireland).

### Eligibility criteria {10}

The eligibility criteria for patients to enrol in this study are as follows.

Inclusion criteria

- Male and female aged > 18
- Able to provide written informed consent
- ASA grade I – V
- MITS surgery
- Weight > 55kg

Exclusion criteria

- Absence of or inability to give informed consent
- Pre-existing infection at block site
- Severe coagulopathy
- Allergy to local anaesthesia (or another contraindication to block performance)
- Previous history of opiate abuse
- Pre-existing chronic pain condition
- Pre-existing dementia (due to need to co-operate in completing QoR-15 score day after surgery
- Postoperative admission to ICU for continued ventilation
- BMI > 40 kg/m^2^

### Who will take informed consent? {26a}

Potential participants for this trial will be identified by a member of the surgical, anaesthetic or research team. These patient’s electronic medical records will be analysed by a member of the research team to determine if they are suitable candidates for this trial, i.e. if they meet the inclusion criteria and have no reason for exclusion.

Suitable patients will be approached the evening before surgery where possible. Alternatively, patients will be approached on the ward on the morning of surgery and their suitability to participate in the trial will be confirmed. The purpose of the trial including benefits and risks, and method of follow up will be explained to patients. A comprehensive and instructive information leaflet will be given to each patient, and they will be provided an adequate amount of time to study it (minimum 10 min). Patients will be informed that their participation in the study is entirely voluntary and they will have the opportunity to withdraw from the study at any time, and this will not affect the quality of care they receive. Finally, a member of the research team will obtain informed written consent from the participant.

### Additional consent provisions for collection and use of participant data and biological specimens {26b}

Not applicable. No data and biological specimens will be collected for use in ancillary studies.

### Interventions

#### Explanation for the choice of comparators {6b}

Erector spinae plane blocks have been embraced by many anaesthesiologists; however, to the best of our knowledge, there are no clinical effectiveness trial comparing infusion techniques of programmed intermittent bolus vs standard continuous infusion for minimal invasive thoracic surgery in terms of patient centred outcomes and therefore this clinical trial is warranted.

#### Intervention description {11a}

Eligible study participants will receive an ultrasound-guided ESP block post induction of general anaesthesia and prior to commencement of surgery. Blocks will be performed under full asepsis with patients in the lateral decubitus position. Initial bolus of the ESP block will be the same in all patients: Twenty millilitres of 0.25% levobupivacaine will be injected at a level corresponding to the mid-point of the range of surgical dermatomes likely to be affected by the surgery. After administration of the bolus dose, an epidural catheter will be advanced 3–5 cm into the erector spinae space. Typically, ESP block and catheter insertion will be administered at T4 level. The ESP block will be performed as follows.

First, the approximate midpoint of the intended surgical incision will be identified. The ultrasound transducer (SonoSite HFL 50x, SonoSite Inc.) will then be placed in a longitudinal orientation approximately to 2–3 cm lateral to the midline, to identify the hyperechoic line of the transverse process with its associated acoustic shadow. After identification of trapezius, rhomboid major and erector spinae muscle groups superficial to the transverse process, an 18G Tuohy epidural needle (Ultraplex; B. Braun, Hessen, Germany) will be advanced in a cranio-caudal direction. The needle tip will be advanced until it is in the interfascial plane deep to the erector spinae muscle group and superficial to the transverse process. Once in position, 20 ml 0.25% levobupivacaine will be injected under ultrasound guidance. Correct needle tip position will be confirmed by the presence of linear spread between the transverse process and the erector spinae muscle group. After administration of a bolus dose, a 20G polyether epidural catheter (Ultraplex 360 cannula; B. Braun, Hessen, Germany; 50e80 mm) will be advanced through the Tuohy needle and into the ESP space. The catheter will be adjusted so that approximately 3–5 cm of the catheter will be in the ESP space. The catheter will then be secured in this position. Because patients will be under general anaesthesia during the block performance, no formal dermatomal sensory testing of block efficacy will be performed at this time. Surgery will begin once the block is completed. There will be no further intervention to the routine conduct of surgery and anaesthesia after this point. Blocks will be performed by anaesthesiologist with subspeciality training in regional anaesthesia. Two hours post administration of the ESP bolus dose, the ESP catheter will be connected and commenced with either the PIB or CI regimen (randomised process).

The PIB patients will receive levobupivacaine 0.125% programmed bolus 20 ml 2 hourly. The CI group will receive continuous infusion levobupivacaine 0.125% at 10 ml.h^−1^. Therefore, both arms of the study will receive 25mg levobupivacaine every 2 h.

#### Criteria for discontinuing or modifying allocated interventions {11b}

Criteria for discontinuing study protocol are as follows:

1. Unexpected regional anaesthesia complication
2. Suspected or confirm diagnosis of local anaesthesia toxicity
3. Unexpected postoperative admission to intensive care unit
4. Inability to insert ESP catheter
5. Patient request to be withdrawn from study
6. Clinical concern of patient’s care from surgical, anaesthesia, or research team

#### Strategies to improve adherence to interventions {11c}

**Fig. 2:**
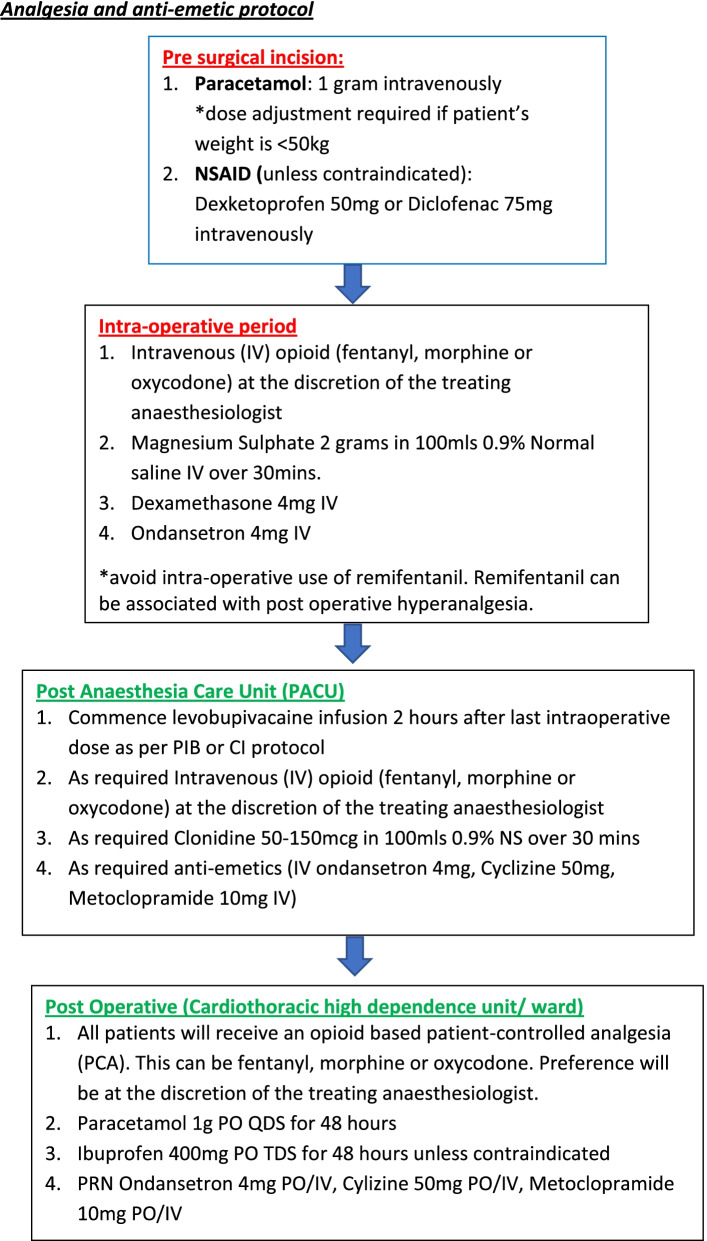
Analgesia and anti-emetic protocol

The study flow chart (Fig. 1) and analgesia and anti-emetic protocol (Fig. 2) will be made available in the operating theatre and post anaesthesia care unit (PACU). These protocols will be available to anaesthesiologist and surgeons performing the intervention and data collectors to ensure protocol adherence.

#### Relevant concomitant care permitted or prohibited during the trial {11d}

All study participants will undergo general anaesthesia (GA) as per standard care for MITS. Induction of GA will be conducted or supervised by a consultant anaesthesiologist. Induction of GA will be achieved intravenous administration of fentanyl, propofol, and a neuromuscular blockade agent at the discretion of the anaesthesiologist.

Airway management will be at the discretion of the treating anaesthesiologist. Ventilation strategy, choice of intraoperative monitoring, and vascular access will also be at the discretion of the treating anaesthesiologist. The haemodynamic goal will be to maintain systolic blood pressure within 20% of the patient’s baseline. Persistent intraoperative elevations above this point will trigger the administration of intravenous opioids. The dosage and timing of this will be at the discretion of the treating anaesthesiologist. Intravenous (IV) paracetamol and an IV non-steroidal anti-inflammatory drug will be delivered intraoperatively where clinically appropriate and at the discretion of the treating anaesthesiologist.

A concomitant care plan for perioperative analgesia and anti-emetics will be standardised for the pre-surgical, intra-operative, PACU, and post-operative period. This is outlined in Fig. 2.

#### Provisions for post-trial care {30}

Study participants are covered by indemnity for negligent harm, through the standard HSE (Health Service Executive) indemnity arrangements. If any complications arise directly from either intervention, the participant will receive standard post-operative management which may include management from the surgical team, pain medicine department, and multidisciplinary team. Additionally, all investigators will be employees of the respective hospitals and will be covered by the HSE clinical indemnity scheme.

### Outcomes {12}

#### Primary outcome measures

1. Quality of Recovery (QoR-15) score of the programmed intermittent bolus group vs the continuous infusion group at 24 h postoperative [time frame: 24 h postoperative] [14, 15].
  - QoR-15 is a 15-parameter questionnaire which has been validated as an optimal tool to measure overall patient recovery after surgery including postoperative pain. Participants will complete this questionnaire at 24 h after their surgery. Each question will be scored from 0 ‘none of the time’ to 10 ‘all the time’ except for questions 11–15, which will be score inverted: 10 ‘none of the time’ to 0 ‘all the time.’ The total QoR-15 score ranges between 0 and 150, where 150 illustrates that the patient has had an excellent recovery.

#### Secondary outcome measures

1. Quality of Recovery (QoR-15) score of the programmed intermittent bolus group vs the continuous infusion group at 48 h postoperative [time frame: 48 h postoperative] [14, 15].
2. Pulmonary function assessment [time frame: pre-operatively day 0, post-operative day 1, and post-operative day 2]
  - Pulmonary function assessment will be undertaken pre-operatively (day 0) before induction of GA and at post-operative day 1 and 2 using bedside incentive spirometry. The average of three maximum inspiration volumes will be measured with the patient in the sitting position. Any change in maximum inspiratory volumes between pre-operative and postoperative days 1 and 2 will be evaluated.
3. Area under the curve (AUC) of Verbal Rating Score (VRS) for pain at rest and on deep inspiration versus time over 48 h [time frame: 60–120 min in PACU, 24 and 48 h post-operative]
  - Verbal Rating scale is measured from 0 to 10, where ‘0’ indicates no pain and ‘10’ indicates severe pain.
4. Time to administration of first postoperative intravenous opioid
5. Duration of time in PACU
6. Total 24 h and 48 h opioid consumption
7. Time to first mobilisation
8. Documentation of adverse events
  - This includes intra-operative haemodynamic changes, post-operative hypotension, nausea and vomiting, pruritis, block failure, and block-related complications.
9. Length of hospital stay [time frame: 1 month]

### Participant timeline {13}

**Fig. 3:**
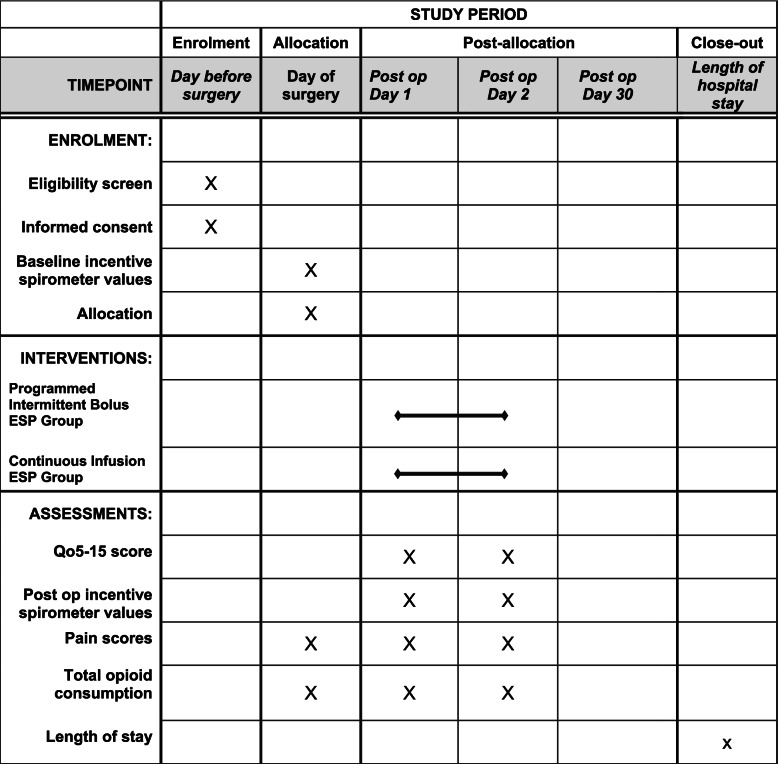
Schedule of enrolment, interventions, and assessments

The schedule of enrolment, interventions, and assessments is outlined below in Fig. 3.

#### Sample size {14}

The primary outcome of this study will be the QoR-15 score at 24 h postoperatively. This score rates 15 subjective, patient-reported parameters about their recovery on a scale of 0–10. Therefore, the minimum possible score is 0, and the maximum possible score is 150. The established minimum clinically important difference in QoR-15 is 6.0 [14], and the mean SD of QoR-15 scores after major surgery is in the order of 8–16. Taking an SD of 8, and assuming type I error = 0.05 and type II error = 0.2 (80% power to detect this difference), then *n* = 28 patients will be required in each group. To accommodate for participants who may withdraw from the study, we will aim to recruit *n* = 30 patients to each study arm, i.e. *n* = 60 in total.

#### Recruitment {15}

Members of the anaesthetic and surgical research team from each clinical site will engage in the recruitment process.

### Assignment of interventions: allocation

#### Sequence generation {16a}

Participants will be randomised to either PIB vs CI group by using an online computer-generated block randomisation (https://www.sealedenvelope.com). Block randomisation will occur in groups of 6 to ensure each arm of the study has an even number of participants. The trial investigators will not have access to the randomisation key/seed until completion of the study.

#### Concealment mechanism {16b}

The participant study number and group allocation will be typed onto separate pages and concealed in sequentially numbered, opaque, sealed envelopes. The randomisation process will be performed by an independent third party who is not involved in the conduction of this trial.

#### Implementation {16c}

After confirming that informed consent for participation in this trial has been signed by the participant, a sealed envelope will be opened by the treating anaesthesiologist to reveal the group allocation. This process will occur *after* induction of general anaesthesia to ensure the patient is blinded to the study intervention.

### Assignment of interventions: blinding

#### Who will be blinded {17a}

This study will be a double-blinded clinical trial. Patients will be blinded to the study because they will receive the intervention after they have been put under general anaesthesia. Members of the research team involved in data collection and analysis will be masked to group allocation. The treating anaesthesiologist and surgeon will not be blinded.

#### Procedure for unblinding if needed {17b}

Allocation of a participant will be revealed immediately if there is a clinical concern, i.e. if the patient met criteria for discontinuing study protocol (part 11b).

### Data collection and management

#### Plans for assessment and collection of outcomes {18a}

Data collection will occur at three distinct time points (pre-operative, intra-operative, and post-operative). Data will be collected from both electronic and paper patient records and directly from the patient by means of completing a questionnaire. The same members of the research team (blinded to the intervention) will collect all peri-operative data. Prior to participant enrolment, data collectors will receive specific training from the principal investigator (DB), regarding data handling, collection, and storage; case record form; and QoR-15 questionnaire completion process. This will ensure high quality data collection during the clinical trial. After completion of patient recruitment, data will be transcribed onto Microsoft Excel, and subsequently, the raw data sheet will be thoroughly investigated by the principal investigator for any accidental input of duplicate measurements and/or for any missing key data points. Furthermore, the principal investigator will be blinded to the patient group and groups will only be unblinded after completion of patient recruitment and statistical analysis of the raw data is ready.

#### Plans to promote participant retention and complete follow-up {18b}

Study participants will receive a comprehensive patient information leaflet (PIL) about the trial. A member of the research team will explain this PIL, study set-up, and the study interventions. The importance of completion of postoperative follow-up will be stressed to all participants.

#### Data management {19}

All patient data collected will be handled in accordance with European Union General Data Protection Regulations (EU 2016/679). Initially, data will be collected manually and then transcribed onto Microsoft Excel. Data collected in each clinical site will be stored securely on a password-protected desktop computer, stored in a locked office in the Department of Anaesthesiology at the respective hospital, such that only investigators assigned to data input, processing, and analysis will have access. Data will be collected directly from source documents into the de-identified encoded paper case record form (CRF) and subsequently entered into the electronic CRF. In accordance with local and international regulations, a copy of the original hardcopy CRF will be stored in a locked cabinet/office accessible to authorised personnel only.

#### Confidentiality {27}

All research data will be stored using a study identification number for each patient. An identifiable patient data page reporting the assigned patient identification code will be stored separately also in a locked cabinet/office (accessible to authorised personnel only) in order to record in-hospital outcomes, supply missing data points and to allow potential monitoring visits by national coordinating investigators. This data page will only be made available to members of the research team responsible for data input and the principal investigator. No patient identification details will be reported in any future publications.

#### Plans for collection, laboratory evaluation, and storage of biological specimens for genetic or molecular analysis in this trial/future use {33}

Not applicable, no samples will be collected.

### Statistical methods

#### Statistical methods for primary and secondary outcomes {20a}

The collected raw data will be inspected for any errors. These include but are not limited to, double entry errors, missing data, and data that was incorrectly entered. Data will be recorded in Excel™ (Microsoft, Redmond, WA, USA) and subsequently imported into GraphPad Prism version 9.3.1 (GraphPad, Salt Lake City, UT, USA) for statistical analysis. All data will be stored according to EU Directive 2019 on General Data Protection Regulations. Data will be inspected and tested for normal distribution according to the Shapiro-Wilk test and quantile-quantile (QQ) plot graph as appropriate. Normally distributed data will be compared between study arms using the unpaired *t* test, whereas non-normally distributed data will be compared using Mann-Whitney *U* test. Fisher’s exact test was used for comparing categorical data. All data will be summarised as mean (SD), median (25–75% range), and *n* (%) as appropriate, and *p* value < 0.05 will be considered statistically significant.

#### Interim analyses {21b}

There are no interim analyses planned.

#### Methods for additional analyses (e.g. subgroup analyses) {20b}

There are no subgroup analyses planned.

#### Methods in analysis to handle protocol non-adherence and any statistical methods to handle missing data {20c}

Our expectation is that very few patients will be lost to follow-up during their inpatient stay due to protocol adherence strategies as mentioned above. Therefore, we expect missing data will be at a minimum when analysing the primary outcome. Multiple imputation will be used if a statistical method is needed to account for missing data in terms of secondary outcomes.

#### Plans to give access to the full protocol, participant-level data, and statistical code {31c}

The assembled data collected will be retained for a maximum 5 years after completion of analysis. A completely de-identified data set will be provided where appropriate upon reasonable request and in agreement with the principal investigator and data protection officer.

### Oversight and monitoring

#### Composition of the coordinating centre and trial steering committee {5d}

The study steering committee will meet monthly to assess progress, address any ongoing organisational or logistical issues, and to consider any adverse effects. A research leader for each clinical site will provide monthly reports to the principal investigator.

#### Composition of the data monitoring committee, its role and reporting structure {21a}

A data protection impact assessment (DPIA) screening tool for this study was completed and analysed by the hospital’s data protection officer (DPO). The decision of the DPO was that no data monitoring committee (DMC) was required for this study as it poses a low risk to the rights and freedoms of natural persons. Therefore, a formal DPIA was not required. Furthermore, a DMC was not appointed, due to the expected expeditious inclusion of participants to this trial. Data collection is expected to be completed in nine to twelve months and minimal inherited risks are associated with this trial.

#### Adverse event reporting and harms {22}

Any unexpected complications which may arise during this trial will be documented and reported to the principal investigator, attending anaesthesiologist, surgical consultant, and to the relevant hospital patient safety officer.

#### Frequency and plans for auditing trial conduct {23}

During this study, an initial auditing process may be conducted using a risk-based approach. This would initially involve focusing on the hospital which may have the largest number of enrolment and/or lost to follow-up rates. The auditing process would include exploring datasets and analysing them for accuracy, missing data, duplicate data and adherence to data protection guidelines. This process would be conducted by an independent reviewer with no involvement with this trial (e.g. research nurse who is affiliated with the clinical site but is not involved with this specific trial).

A research nurse affiliated with the department of anaesthesia at MMUH but who is not involved in this trial may undertake an audit involving the exploration of datasets from both institutions. This would be precipitated by risk indices including high rates of dropout rates.

#### Plans for communicating important protocol amendments to relevant parties (e.g. trial participants, ethical committees) {25}

We define a substantial modification of the study protocol as changes which may affect the outcome of the study or patient safety. Such changes include the following: any modification to the aims of the study, study design, and the inclusion or exclusion criteria or any alternations of the study interventions (e.g. using new procedural equipment or conducting any intervention which deviates from the original description). Any amendment will be agreed upon the principal investigator of this trial and will seek approval from the Ethics Committee/IRB. Minor changes of the protocol include any administrative changes or alternation of the analgesia plan that do not impact patient safety nor the conduct of the trial (e.g. changes to anti-emetic medications). The Ethics Committee/IRB may be notified of minor changes at the discretion of the principal investigator.

#### Dissemination plans {31a}

The results from this clinical trial will be fully disclosed by means of publication in an international peer-reviewed journal and by presentation at national and international scientific meetings. Both positive and negative results will be disclosed.

## Discussion

In conducting this randomised control trial, we aim to investigate the efficacy, in terms of quality of recovery, of erector spinae plane catheters using programmed intermittent bolus regimes compared to continuous infusion regimes. Fascial plane blocks including the ESP block have become increasingly popular in recent times, and their use has been reported in a variety of thoracic, spinal, and abdominal surgeries fascial where they may have a role in terms of improving postoperative pain relief, quality of recovery, and patient satisfaction [6, 16–18]. The increase in popularity in fascial plane blocks could be due to a combination of enhanced ultrasound technology and subsequently better understanding of the sonoanatomy of fascial planes and surrounding structures [19]. A fascial plane block is a regional anaesthesia technique in which the space between two fascial layers is the target. The major advantage of fascial plane blocks when compared to neuraxial anaesthesia techniques are their ease of performance and better inherent safety profile. While the exact mechanism of action of ESP is the subject of ongoing debate, its analgesic effects for lumbar surgery are believed to derive from the spread of the local anaesthetic agent to the dorsal and ventral rami of spinal nerves via paravertebral spread-by-proxy [20]. Single-shot truncal regional anaesthesia techniques are restricted by the limited duration of analgesia; catheter techniques can offer the potential benefit of prolonged analgesia. Previous studies using catheter techniques for MITS have used the traditional end points of opioid consumption and pain scores to assess efficacy [21]. The VAS is an imperfect scale without psychometric evaluation that overlooks the individual components of recovery and is prone to overrating [15]. Defining success in regional anaesthesia is multifactorial, and using patient-centred, population-centred, healthcare-centred, and training-centred outcomes may be more beneficial. The QoR-15 can be an advantageous outcome measure in clinical trials and for assessing the impact of changes in health care delivery for quality assurance purposes [15]. To the best of our knowledge, there are no randomised controlled trials comparing continuous infusion versus intermittent bolus strategies for erector spinae plane block for MITS in terms of patient-centred outcomes such as quality of recovery.

Bolus injection of a local anaesthetic followed by continuous infusion has been the standard technique for fascial plane catheters such as PVB and ESP for post-thoracotomy analgesia [7, 22]. However, it has been shown in paravertebral blocks that the range of anesthetised dermatomes becomes gradually narrower when the local anaesthetic is administered at a constant rate [23]. Theoretically, the addition of a bolus injection of local anaesthetic to continuous infusions may maintain the range of anesthetised dermatomes; however, this theory has not been elucidated in clinical trials relating to ESP blocks. In this study, we hypothesise that programmed intermittent erector spinae plane bolus of levobupivacaine will maintain a wider sensory block and improved quality of recovery compared with continuous infusion.

We acknowledge some limitations to this protocol including the fact that the treating anaesthesiologist will not be blinded to the group allocation as they will be required to perform the block. However, the primary will be measured 24 h postoperatively by researchers blinded to the group allocation, thus maintaining blinding of data. We acknowledge that for some patients having MITS their recovery will continue beyond 48 h postop; however, the acute postoperative pain following MITS is diminished by day 3, and many of our patients will be discharged home at this stage. Length of stay and postoperative complications will continue to be observed until patient discharge. ESP catheters will be placed under general anaesthesia; therefore, formal dermatomal assessment of block function will not be possible, and we will not be formally testing block effectiveness. However, the practice of placing these catheters under ultrasound guidance after induction of general anaesthesia is in line with common clinical practice, and therefore, our findings should still be applicable to widespread clinical practice. Assessment of preoperative QoR-15 will not be undertaken in this study. Therefore, we will not have a baseline from which to compare postoperative QoR-15 scores. Nonetheless, QoR-15 was specifically intended for postoperative use, and we will apply this scoring tool equally to both randomised groups. Furthermore, the accuracy of QoR-15 in the immediate pre-operative period has been debated [24]. By focusing on patient-centred outcomes in our trial design, we hope to explicate whether the use of programmed intermittent regimes of delivery of levobupivacaine to the erector spinae plane will result in an improved quality of recovery compared with continuous infusion.

## Trial status

The trial is registered on ClinicalTrials.gov Identifier: NCT05181371. The current protocol is version 2 of 23 May 2022. Participant recruitment commenced on 2 June 2022, and full patient recruitment is estimated to be completed by July 2023.

## Supplementary Information

**Additional file 1.**

## Data Availability

The final trial dataset will be made available from the corresponding and principal author upon reasonable request.

## Abbreviations

MITS: Minimally invasive thoracic surgery
QoR-15: Quality of recovery
CPSP: Chronic persistent surgical pain
PIB: Programmed intermittent bolus
CI: Continuous infusion
VATS: Video-assisted thoracic surgery
RATS: Robotic assisted thoracic surgery
ESP: Erector spinae plane
RCT: Randomised control trial
PVB: Paravertebral block
MMUH: Mater Misericordiae University Hospital
SJH: St James University Hospital
ASA: American Society of Anaesthesiology
ICU: Intensive care unit
PACU: Post anaesthesia care unit
GA: General anaesthesia
HSE: Health Service Executive
AUC: Area under the curve
CCI: Comprehensive Complication Index Calculator
SD: Standard deviation
PIL: Patient information leaflet
CRF: Case record form
DMC: Data monitoring committee
DPIA: Data protection impact assessment
DPO: Data protection officer
