# Supplementary material for: Ultrasound-guided, continuous erector spinae plane (ESP) block in minimally invasive thoracic surgery—comparing programmed intermittent bolus (PIB) vs continuous infusion on quality of recovery and postoperative respiratory function: a double-blinded randomised controlled trial
Source: Trials. 2022 Sep 21;23:792. doi: 10.1186/s13063-022-06726-7 (PMC9490991; doi:10.1186/s13063-022-06726-7)

Office use

Date:

Study number:

Operation:

**Pre-operative/Pre-induction of GA**

| **Variable** | 1 | 2 | 3 | Average |
| --- | --- | --- | --- | --- |
| BP (sys/dia/MAP) |  |  |  |  |
| HR |  |  |  |  |
| Inspiratory incentive Spirometry {sitting position} (ml) |  |  |  |  |

| ASA |  |
| --- | --- |
| Gender |  |
| Age |  |
| Weight (kg) |  |
| Height (cm) |  |
| BMI |  |

| Regional Block Complications at time of insertion | Please state and explain: |
| --- | --- |
| Time to complete Block | Mins |

**Intra-operative**

| **Recovery Room Variable** |  |  |  |  |  |
| --- | --- | --- | --- | --- | --- |
| Time of arrival |  | | | |  |
| Time of departure |  | | | |  |
| Pain at rest | /10 | | | |  |
| Pain at deep inspiration | /10 | | | |  |
| Time to first opioid |  | | | |  |
| Opioid given | Fentanyl IV | Morphine IV | Oxynorm IV | Other opioid | Total Recovery po Morphine equivalent |
|  |  |  |  |  |  |
| Other analgesia: | Please state: | | | |  |
| Hypotension (sys <90mmHg or MAP <60mmHg) | Yes  No  Please Circle | | | |  |
| Anti-emetics given | Please circle: Yes No | | | |  |
| Pruritis | Please circle: Yes No | | | |  |

| **Variable** |  |  |  |  |
| --- | --- | --- | --- | --- |
| Highest intra-op BP (sys/dia/MAP) |  | | | |
| Lowest Intra-op BP (sys/dia/MAP) |  | | | |
| Highest Intra-op HR |  | | | |
| Intra-op Opioids (After Block placement and loading of LA dose) | **Total IV Fentanyl** | **Total IV Morphine** | **Total IV Oxynorm** | **Total intra-op po Morphine Equivalent** |
| Duration of surgery (skin incision-closure) | **mins** | | | |

**Recovery and Post op 24 & 48 hours**

| **Post Op Variables** | **At 24 hours post op** | **At 48 hours post op** |
| --- | --- | --- |
| QoR15 Score | /150 | /150 |
| Pain at rest | /10 | /10 |
| Pain at deep inspiration | /10 | /10 |
| Number of times anti-emetics given |  |  |
| Pruritis complaint | Please circle: Yes No | Please circle: Yes No |
| Inspiratory Spirometry 1^st^ attempt (ml) |  |  |
| Inspiratory Spirometry 2^nd^ attempt (ml) |  |  |
| Inspiratory Spirometry 3^rd^ attempt (ml) |  |  |
| Inspiratory Spirometry Average (ml) |  |  |
| Total opioid received  (fentanyl PCA and/or Po Oxynorm) |  |  |
| Total PO Morphine equivalent (mg) |  |  |
| Sensory Level of Block | (e.g. T4-T9) |  |
| Dermatomes of surgical incision |  |  |
| Motor Block present | Please circle: Yes No | Please circle: Yes No |
| Block Failure | Please circle: Yes No | Please circle: Yes No |
| Block complication | If yes please state: | If yes please state: |
| Time Post op to first mobilization |  |  |
| Time to first post op opioid if none given in PACU |  |  |

**Retrospective analysis once patient is discharged**

| Length of hospital stay | days |
| --- | --- |

**Q0R15 24 hours** **QoR15 48 hours**


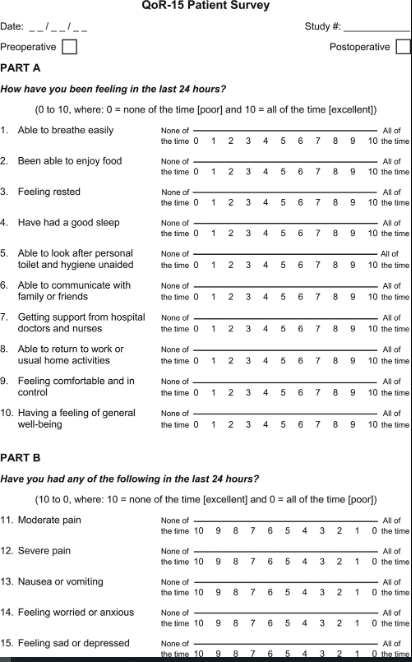

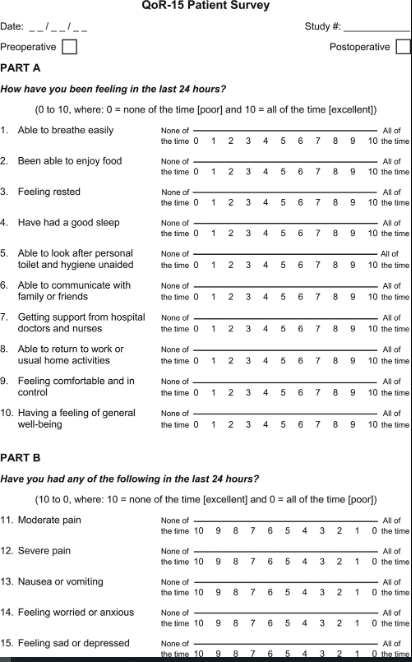

Supplement: Supplementary file 1 — Additional file 1. [file 13063_2022_6726_MOESM1_ESM.docx]
